# Neuromonitoring of delirium with quantitative pupillometry in sedated mechanically ventilated critically ill patients

**DOI:** 10.1186/s13054-020-2796-8

**Published:** 2020-02-24

**Authors:** Eva Favre, Adriano Bernini, Paola Morelli, Jerôme Pasquier, John-Paul Miroz, Samia Abed-Maillard, Nawfel Ben-Hamouda, Mauro Oddo

**Affiliations:** 1 Critical Care Research Unit, Centre Hospitalier Universitaire Vaudois (CHUV), University Hospital and University of Lausanne, Rue du Bugnon 46, BH08-623, CH-1011 Lausanne, Switzerland; 2 Department of Intensive Care Medicine, Centre Hospitalier Universitaire Vaudois (CHUV), University Hospital and University of Lausanne, Rue du Bugnon 46, BH 08.623, Lausanne, Switzerland; 3 Institute of Higher Education and Research in Healthcare – IUFRS, University of Lausanne, Lausanne, Switzerland; 4 Center for Primary Care and Public Health, University of Lausanne, Lausanne, Switzerland

**Keywords:** Delirium, Pupillometry, Cholinergic, Pupillary reactivity, Mechanical ventilation

## Abstract

**Background:**

Intensive care unit (ICU) delirium is a frequent secondary neurological complication in critically ill patients undergoing prolonged mechanical ventilation. Quantitative pupillometry is an emerging modality for the neuromonitoring of primary acute brain injury, but its potential utility in patients at risk of ICU delirium is unknown.

**Methods:**

This was an observational cohort study of medical-surgical ICU patients, without acute or known primary brain injury, who underwent sedation and mechanical ventilation for at least 48 h. Starting at day 3, automated infrared pupillometry—blinded to ICU caregivers—was used for repeated measurement of the pupillary function, including quantitative pupillary light reflex (q-PLR, expressed as % pupil constriction to a standardized light stimulus) and constriction velocity (CV, mm/s). The relationship between delirium, using the CAM-ICU score, and quantitative pupillary variables was examined.

**Results:**

A total of 59/100 patients had ICU delirium, diagnosed at a median 8 (5–13) days from admission. Compared to non-delirious patients, subjects with ICU delirium had lower values of q-PLR (25 [19–31] vs. 20 [15–28] %) and CV (2.5 [1.7–2.8] vs. 1.7 [1.4–2.4] mm/s) at day 3, and at all additional time-points tested (*p* < 0.05). After adjusting for the SOFA score and the cumulative dose of analgesia and sedation, lower q-PLR was associated with an increased risk of ICU delirium (OR 1.057 [1.007–1.113] at day 3; *p* = 0.03).

**Conclusions:**

Sustained abnormalities of quantitative pupillary variables at the early ICU phase correlate with delirium and precede clinical diagnosis by a median 5 days. These findings suggest a potential utility of quantitative pupillometry in sedated mechanically ventilated ICU patients at high risk of delirium.

## Introduction

Delirium is a common neurological complication of intensive care unit (ICU), particularly in patients requiring prolonged mechanical ventilation and sedation. Delirium pathophysiology is multifactorial, involving abnormalities of microcirculatory and endothelial function, neurotransmitter imbalance, increased cytokine release and activation of neuroinflammation [1, 2]. The autonomous nervous system, through cholinergic activation, has an innate counter-regulatory role against increased neuro-inflammation [3], via an inhibition of cytokine release and increased inflammatory neurotransmitters [4–7]. Growing evidence demonstrates that reduced functioning of the cholinergic anti-inflammatory reflex is implicated in the pathophysiology of secondary critical illness-related brain dysfunction [8], which in turn can be attenuated by therapeutic vagus nerve stimulation [9–13].

The pupillary light reflex (PLR) is regulated by the cholinergic system, which mediates pupillary constriction to light stimulation [6]; therefore, quantitative measurement of the pupillary function with automated infrared pupillometry represents an attractive tool for evaluating cholinergic activity in the clinical setting [14–18] and is emerging as a novel monitoring and diagnostic tool in neurological conditions (e.g., Alzheimer’s and Parkinson’s disease) in which cholinergic deficit is implicated in disease pathogenesis [19–21].

Composite prediction scores (e.g., E-PRE-DELIRIC [22] and PRE-DELIRIC [23]) may help in improving delirium prediction [24]; however, there are currently no available quantitative tools for the monitoring of ICU delirium. The objective of this study was to examine in high-risk sedated mechanically ventilated patients, without primary acute or known brain injury, whether reduced pupillary light constriction—assessed quantitatively at the early phase of ICU using automated infrared pupillometry—was associated with delirium, assessed with the Confusion Assessment Method for the ICU (CAM-ICU) [25].

## Materials and methods

### Study population

This was a prospective observational cohort study conducted from December 2016 to March 2018 at the Department of Adult Intensive Care Medicine, Centre Hospitalier Universitaire Vaudois (CHUV), University Hospital and University of Lausanne, Switzerland. Subjects were medical-surgical critically ill patients requiring sedation and mechanical ventilation for at least 48 h, at high risk (about 50%) of ICU delirium [26]. Exclusion criteria were mechanical ventilation for ≤ 48 h, age < 18 years, acute brain injury (including traumatic brain injury, ischemic/hemorrhagic stroke, subarachnoid hemorrhage, hypoxic-ischemic brain injury after cardiac arrest, meningo-encephalitis, status epilepticus, hepatic encephalopathy, neurosurgical intervention), previous known cognitive impairment, end-stage renal or hepatic disease (Child-Pugh B and C cirrhosis), pregnancy, ICU readmission, or transfer from another ICU. Additional exclusion criteria included a pre-existing ophthalmic condition or disease that may alter pupillary response, including cataract surgery, multiple sclerosis, amyloidosis, sclerodermia, and multiple system atrophy. Patients who were expected to die within 72 h were also excluded. A convenience sample size was used (*n* = 100). The study was approved by the ethical committee of the Lausanne University (project-ID 2016-01923), and a waiver of consent was granted because non-invasive pupillometry is standard care. The study conforms with the STROBE guidelines for the report of observational studies.

### Management of analgesia and sedation

Management of analgesia and sedation was based on a written institutional algorithm, in line with current recommendations [27]. Sedation was targeted to Richmond Sedation Agitation Scale (RASS) [28] with continuous infusions of propofol (2–3 mg/kg/h) and/or midazolam. Propofol was generally first-line agent; midazolam was used in patients with hemodynamic instability (defined as norepinephrine > 0.25 μg/kg/min), or when the propofol dose exceeded 4 mg/kg/h or was maintained for more than 48 h. Analgesia was maintained with fentanyl (1–1.5 μg/kg/h).

### Automated infrared pupillometry

An automated infrared pupillometer (AlgiScan®, ID-Med, Marseille, France) was used for repeated measurements of quantitative PLR (q-PLR; expressed as the percentage change of pupillary diameter following the light stimulus) and constriction velocity (CV; measuring the speed of pupil constriction following light stimulation, expressed in mm/s). Normative values for the q-PLR range between 30 and 40%, and for the CV between 1.5 and 2.2 mm/s; low values are defined as a q-PLR < 20% and a CV < 1 mm/s, respectively [29].

Pupillary measurements were conducted on both eyes by an experienced clinician or nurse, who was not involved in patient care, and were performed during the day in stable conditions of ambient light. Measurements were performed twice daily (in the morning and the afternoon), starting at day 3 from mechanical ventilation, and were repeated at days 4 and 5, up to a maximum of day 7. At each time-point, the average values of q-PLR and CV from both eyes were retained for the analysis. All pupillometry variables were blinded to clinicians and nurses involved in patient care.

### Outcome assessment

As soon as the Richmond Agitation-Sedation Score was equal or greater than − 2, delirium was assessed twice daily until discharge from the ICU using the CAM-ICU. Patients were considered as having delirium when they had at least one positive CAM-ICU during their ICU stay. The duration of coma was calculated as the number of days spent with a motor Glasgow Coma Scale < 6 from ICU admission.

### Data processing and statistical analysis

Demographic and clinical variables included age, gender, medical versus surgical ICU admission, admission APACHE II score, daily SOFA score, sepsis diagnosis (according to the Sepsis-3 definition [30]), cumulative dose of analgesia (fentanyl) and sedatives (midazolam and propofol) during the first 7 days of ICU, duration of mechanical ventilation, length of ICU stay, and 90-day mortality.

Data are presented as median and interquartile range (IQR), except when otherwise stated. Univariate comparisons between the two main outcome groups (delirium vs. no-delirium) were analyzed using the non-parametric Wilcoxon test. A multivariable stepwise logistic regression model was performed by entering the day 3 q-PLR as the variable of interest, and the day 3 SOFA score, the cumulative fentanyl dose, and the cumulative dose of sedatives as pre-specified co-variates. Statistical significance was set at *p* < 0.05. Statistical analysis was conducted with R 3.5.1 and JMP-14®. The statistical analysis was performed by an independent statistician (JP).

## Results

### Patient characteristics

A total of 100 patients were studied. Patient demographics are summarized in Table 1. The majority of patients had sepsis (76%). Delirium prevalence was 59%, and median time from ICU admission to delirium diagnosis was 8 days (IQR 5–13); 3 out of 100 patients died in the ICU, previous to CAM-ICU assessment, and were therefore not included in the final analysis. Mortality at 90 days was 10%.

**Table 1:** Patient demographics

Variable	Value
Patient number	100
Female gender, *n* (%)	67 (67)
Age, years	65 (53–74)
APACHE II score	22 (17–27)
SOFA score	12 (9–14)
Primary ICU admission	
Pneumonia	24 (24)
Peritonitis	23 (23)
Cardiovascular surgery	11 (11)
Chronic obstructive pulmonary disease	11 (11)
Hemorrhagic shock	9 (9)
Heart failure	8 (8)
Pancreatitis	6 (6)
Mediastinitis	4 (4)
Polytrauma	3 (3)
Medical admission, *n* (%)	42 (42)
Surgical admission, *n* (%)	58 (58)
Sepsis, *n* (%)	76 (76)
Duration of coma*, days	5 (2–10)
Duration of mechanical ventilation, days	7 (5–14)
ICU delirium**, *n* (%)	57 (59)
Days from ICU admission to delirium diagnosis	8 (5–13)
ICU length of stay, days	13 (9–20)
90-day mortality, *n* (%)	10 (10)

Data are presented as median (25th–75th percentiles) or percentage (%)

Abbreviations: *APACHE* Acute Physiology and Chronic Health Evaluation, *SOFA* Sequential Organ Failure Assessment

*Duration of coma was defined as the number of days from ICU admission with a Glasgow Coma Scale-motor response < 6; **ICU delirium was evaluated twice daily with the CAM-ICU assessment; 3/100 patients died before CAM-ICU evaluation

### Associations between ICU delirium and quantitative pupillary variables

As shown in Table 2, patients with ICU delirium had higher SOFA score and received a greater 7-day cumulative dose of continuous infusions of midazolam and fentanyl, as compared with subjects without delirium. Patients with ICU delirium had also significantly longer duration of coma, mechanical ventilation, and ICU stay.

**Table 2:** Univariate comparisons between patients with and without delirium

Variable	Delirium (*N* = 57)	No delirium (*N* = 40)	*P* value
Age, years	66 (55–75)	63 (53–73)	0.48
Female gender, *n* (%)	17 (30)	16 (40)	0.38
Medical admission, *n* (%)	25 (44)	16 (40)	0.83
Sepsis, *n* (%)	45 (79)	29 (73)	0.48
APACHE II score, *n*	22 (17–28)	20 (17–24)	0.18
SOFA score, *n*	12 (10–14)	11 (8–12)	0.01
Midazolam, mg/kg*	3.0 (1.5–5.6)	1.2 (0.2–4.0)	0.02
Propofol, mg/kg*	125.6 (24.9–238.4)	94.4 (51.9–278.2)	0.90
Fentanyl, μg/kg*	109.2 (71.8–149.3)	80.2 (44.1–131.3)	0.09
Duration of coma, days	7 (4–10)	4 (1–6)	0.003
Duration of mechanical ventilation, days	10 (6–20)	6 (4–8)	0.003
ICU length of stay, days	14 (11–25)	10 (6–14)	0.001
90-day mortality, *n* (%)	3 (5)	4 (10)	0.44

Data are presented as median (25th–75th percentiles) or percentage (%)

*First 7 days cumulative dose received by continuous infusion

**Fig. 1:**
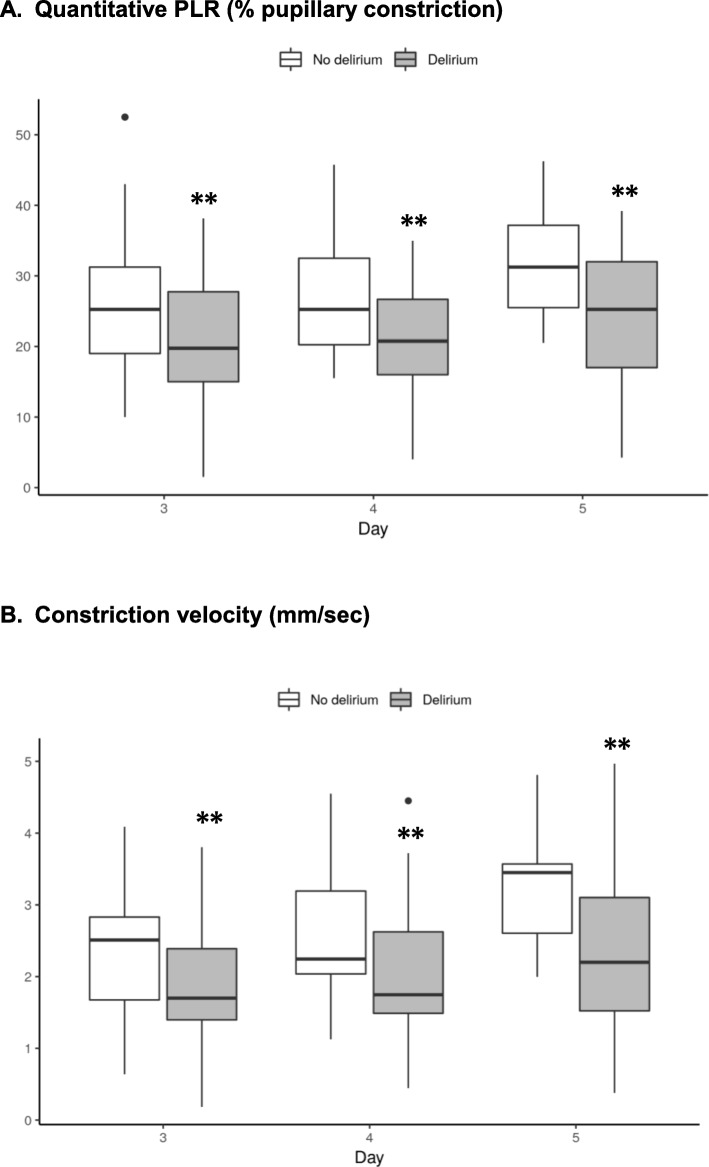
Trends over time of quantitative pupillary light reflex (PLR) and constriction velocity in patients with and without delirium. ** *p* < 0.05 for comparison between groups. **a** Quantitative PLR (% pupillary constriction). **b** Constriction velocity (mm/s)

Compared to non-delirious subjects, patients with ICU delirium had lower values of q-PLR and CV, at all time-points tested, starting at day 3 from mechanical ventilation, and up to day 5 (all *p* < 0.05 for comparisons, Table 3). Trends over time of q-PLR and CV in delirious vs. non-delirious patients are illustrated in Fig. 1. In the subset of patients in whom pupillometry measurements were performed at days 6 and 7 (*n* = 30), q-PLR remained significantly lower in patients with delirium (median 22 [19–33] vs. 30 [29–43] in non-delirious patients at day 6, and 27 [25–31] vs. 35 [27–38] at day 7; both *p* < 0.05).

**Table 3:** Associations between pupillometry variables and delirium

Pupillometry variables	Delirium	No delirium	*P* value
DAY 3			
Pupil size, mm	2.4 (2.2–2.9)	2.9 (2.3–3.4)	0.137
Quantitative PLR, % pupil constriction	20 (15–28)	25 (19–31)	0.012
Constriction velocity, mm/s	1.7 (1.4–2.4)	2.5 (1.7–2.8)	0.017
DAY 4			
Pupil size, mm	2.7 (2.3–3.2)	3.0 (2.5–4.0)	0.045
Quantitative PLR, % pupil constriction	21 (16–27)	25 (20–33)	0.007
Constriction velocity, mm/s	1.7 (1.5–2.6)	2.2 (2.0–3.2)	0.014
DAY 5			
Pupil size, mm	3.1 (2.5–3.8)	3.8 (3.2–4.3)	0.011
Quantitative PLR, % pupil constriction	25 (17–32)	31 (25–37)	0.008
Constriction velocity, mm/s	2.2 (1.5–3.1)	3.5 (2.6–3.6)	0.009

Data are presented as median (25th–75th percentiles)

Abbreviations: *CV* constriction velocity, *PLR* pupillary light reflex

### Reduced quantitative PLR at day 3 is a significant risk factor of ICU delirium

By multivariable analysis, after adjusting for the SOFA score and the cumulative dose of midazolam and fentanyl, reduced q-PLR at day 3 was associated with an increased likelihood of ICU delirium (odds ratio 1.057; 95% confidence interval 1.007–1.113, *p* = 0.03) (Table 4).

**Table 4:** Multivariable logistic regression with independent risk factors for ICU delirium

Variable	Odds ratio	95% confidence interval	*P* value
Quantitative pupillary light reflex	1.057	1.007–1.113	0.03
SOFA score	1.140	0.981–1.337	0.09
Fentanyl dose	1.005	0.999–1.013	0.14
Midazolam dose	0.998	0.863–1.163	0.98

After adjustment for the SOFA score, and the cumulative dose of midazolam and fentanyl, lower quantitative pupillary light reflex at day 3 was associated with an increased risk of ICU delirium

A model combining SOFA score and q-PLR at day 3 yielded an area under the ROC curve of 0.71 for delirium prediction. Of note, independent associations between delirium and q-PLR remained significant at days 4 and 5. No statistically significant interaction was found between q-PLR and the dose of midazolam and fentanyl, at any time-point tested between day 3 and 5 (all *p* > 0.2).

## Discussion

This is the first clinical study investigating the role and potential utility of quantitative pupillometry in sedated mechanically ventilated medical-surgical ICU patients without initial acute or known primary brain injury. In a selected cohort at high prevalence of ICU delirium, we found that delirium was associated with significantly lower values of quantitative PLR and CV, which persisted over time, at the early ICU phase, irrespective of disease severity and analgesia-sedation dose. Our findings have both pathophysiological and clinical implications.

From the pathophysiological standpoint, reduced pupillary constriction in the delirious population, irrespective of age, opioid dose, and disease severity, supports the concept that cholinergic deficit may be a causal factor, thereby establishing a potential pathophysiological basis to our findings [31]. Automated infrared pupillometry may be a valid research tool to investigate autonomous nervous system dysfunction in critically ill patients [32, 33] and may be useful in future studies on ICU delirium.

From the clinical standpoint, our cohort is representative of an ICU population at high-risk for delirium (about 50%), in which there are currently no monitoring tools for early delirium detection [24]. The delirium prevalence was comparable to other reports [26, 34, 35], and as previously observed, delirious subjects had higher cumulative analgesia-sedation dose [36, 37] and greater disease severity [38, 39]. Pupillary constriction is reduced by opioids [40] and sedatives [41]; however, the associations between ICU delirium and reduced q-PLR and CV remained statistically significant after adjusting for opioid and sedation dose, and no significant interaction was found between pupillary constriction variables and fentanyl or midazolam. We found that the best model for predicting delirium, with an area under the ROC curve of 0.71, was the one combining q-PLR and SOFA score at day 3. These findings imply that mechanically ventilated sedated patients with high SOFA score and low q-PLR are at considerably higher risk of ICU delirium. In this patient population, automated infrared pupillometry may be of particular benefit. It is plausible to postulate that abnormally low quantitative pupillary constriction variables may trigger interventions targeted at limiting delirium risk, or complement available composite scores for predicting delirium [22].

### Study limitations

The study was single-center and utilized a convenience sample size, without formal sample size calculation, thereby implying a potential risk of biases. The cohort was selected to be representative of a high-risk ICU delirium population, undergoing mechanically ventilation for at least 48 h or more, i.e., a setting where neuromonitoring may be of greatest potential utility. However, pupillometry was not started early on ICU admission in all patients expected to be on mechanical ventilation for at least 48 h, but rather was restricted to patients who were actually still mechanically ventilated after 48 h. It therefore remains to be investigated whether very early pupillometry assessment may provide even earlier evidence for risk of delirium. The duration of the delirium was not available in all patients, which is an additional limitation.

Neuroimaging was not systematically performed, but we excluded all patients admitted for a primary acute brain injury or with a previous known neurological disease thereby limiting as much as possible intrinsic brain factors that may potentially alter pupillometry assessment [42, 43]. Furthermore, pupillometry measurements were performed by an experienced research ICU physician or nurse, thereby guaranteeing data reliability and quality, and the pupillometry data were blinded to clinicians involved in patient care. We did not adjust for ambient light conditions, which may at least in part affect q-PLR [44]. However, the pupillometer used in this study (AlgiScan® device) has a black rubber that completely covers the eye, thereby ensuring homogeneous dark conditions during pupillary constriction measurements. The average absolute difference in pupil constriction between delirious and non-delirious patients was relatively low—ranging from 0.2 to 0.3 mm—which approaches the limits of inter-rater variability for the device [45]. Additional computed variables such as the Neurological Pupil index (NPi) [40] were not available in this study, but warrants further investigation. Our findings are hypotheses-generating: additional larger, ideally multicenter, studies are needed to confirm our data and more precisely assess the value of low q-PLR in predicting ICU delirium, and identify precise prognostic cutoffs in this setting.

## Conclusions

Automated infrared pupillometry revealed a strong association between lower pupillary light constriction and ICU delirium, irrespective of baseline injury severity and cumulative analgesia and sedation dose. Importantly, alterations in quantitative pupillary constriction variables occurred early and preceded delirium diagnosis by a median 5 days. The findings of this study provide novel insights into ICU delirium pathophysiology and suggest a potential clinical utility of quantitative pupillometry for the neuromonitoring of sedated mechanically ventilated patients at high risk for ICU delirium.

## Data Availability

The datasets used and/or analyzed during the current study are available from the corresponding author on reasonable request.
